# Human Ovarian Follicles Xenografted in Immunoisolating Capsules Survive Long Term Implantation in Mice

**DOI:** 10.3389/fendo.2022.886678

**Published:** 2022-06-03

**Authors:** Margaret A. Brunette, Hadrian M. Kinnear, Prianka H. Hashim, Colleen L. Flanagan, James R. Day, Marilia Cascalho, Vasantha Padmanabhan, Ariella Shikanov

**Affiliations:** ^1^ Department of Biomedical Engineering, University of Michigan, Ann Arbor, MI, United States; ^2^ Program in Cellular and Molecular Biology, University of Michigan, Ann Arbor, MI, United States; ^3^ Medical Scientist Training Program, University of Michigan, Ann Arbor, MI, United States; ^4^ Department of Obstetrics and Gynecology, University of Michigan, Ann Arbor, MI, United States; ^5^ Department of Surgery, University of Michigan, Ann Arbor, MI, United States; ^6^ Department of Microbiology & Immunology, University of Michigan, Ann Arbor, MI, United States; ^7^ Department of Pediatrics & Communicable Diseases, University of Michigan, Ann Arbor, MI, United States

**Keywords:** human ovaries, immunoisolation, poly (ethylene glycol), xenografts, hydrogels

## Abstract

Female pediatric cancer survivors often develop Premature Ovarian Insufficiency (POI) owing to gonadotoxic effects of anticancer treatments. Here we investigate the use of a cell-based therapy consisting of human ovarian cortex encapsulated in a poly-ethylene glycol (PEG)-based hydrogel that replicates the physiological cyclic and pulsatile hormonal patterns of healthy reproductive-aged women. Human ovarian tissue from four donors was analyzed for follicle density, with averages ranging between 360 and 4414 follicles/mm^3^. Follicles in the encapsulated and implanted cryopreserved human ovarian tissues survived up to three months, with average follicle densities ranging between 2 and 89 follicles/mm^3^ at retrieval. We conclude that encapsulation of human ovarian cortex in PEG-based hydrogels did not decrease follicle survival after implantation in mice and was similar to non-encapsulated grafts. Furthermore, this approach offers the means to replace the endocrine function of the ovary tissue in patients with POI.

## 1 Introduction

Over 500,000 survivors of pediatric cancers live in the United States today (1). Due to advances in anticancer therapy, the 5-year survival rate for pediatric cancer patients reached nearly 85% in 2016, which is a significant increase from 50% in the 1970s (1, 2). This population will continue to grow as the incidence rate of pediatric cancers continues to increase, with an estimated 10,500 new diagnoses in 2021 for children aged 0-14 (1). Unfortunately, chemotherapy and radiation can have detrimental effects on male and female gonads, which may result in delayed or completely absent pubertal development (3). The delay or absence of pubertal development negatively impacts patient quality of life and carries long-term health risks associated with decreased bone strength and endocrinopathies (4). The standard of care for puberty induction in adolescent girls with premature ovarian insufficiency (POI) is hormone replacement therapy (HRT), which was developed to treat postmenopausal symptoms in mature women and does not recapitulate physiologic cyclic and pulsatile hormonal patterns found in healthy, reproductive aged women (5, 6). HRT delivers constant doses of estrogen and progesterone, reconstituting ovarian function only partially (7), and precluding ovarian-body homeostasis. Furthermore, the long-term safety and efficacy of HRT to induce puberty has yet to be established (8).

To address the lack of available treatments to induce physiological puberty in adolescent girls with POI, we have developed an immunoisolating hydrogel-based capsule for implantation of donor ovarian tissue without the need for immunosuppression (9–11). Multiple biomaterials have been investigated for supporting follicle growth *in vitro* and *in vivo*, including alginate (12–14), alginate-matrigel (15), fibrin (12, 16), fibrin-alginate (12, 17, 18), and poly(ethylene glycol) (PEG) (19, 20). Here we utilize a system comprised of ovarian tissue surrounded by a degradable PEG hydrogel that promotes follicle growth and expansion. The degradable PEG hydrogel core is surrounded by a non-degradable PEG shell that prevents infiltration of immune cells while allowing diffusion of oxygen, nutrients, and hormones. Our previous studies with these PEG capsules and murine ovarian tissue grafts demonstrated that (1) follicles survive and undergo folliculogenesis for at least 60 days, (2) the capsule prevents immune cell infiltration, and (3) the estrus cycle is restored after encapsulated allogenic tissue is implanted in ovariectomized mice (10).

It remains unknown whether the immunoisolating capsule can support the survival of human ovarian tissue *in vivo*, a key step towards translating this technology to clinical use. Despite many similarities with murine physiology, human ovarian tissue carries some significant morphological differences. Human ovarian tissue is heterogenous with respect to stromal tissue structure, stromal cell and follicle distribution. The majority of primordial follicles are found in the cortex surrounded by a dense stromal tissue, while more mature follicles are found closer to the medulla. In contrast, murine ovarian tissue is more homogeneous, with densely packed follicles present throughout the entire ovary and surrounded by significantly looser stroma (21). Furthermore, follicle density varies between different donors, the right and left ovary from the same donor, and different locations in the cortex from the same ovary (22). Lastly, murine and human primordial follicles are approximately the same size (~30μm), but the terminal diameter of pre-ovulatory follicles in mice is only ~400μm, while human follicles reach a much larger terminal diameter ranging from 2,000 to 20,000μm (23). Keeping these differences in mind, the main objective of this study was to investigate whether follicles in human ovarian tissues survive the encapsulation process and maintain viability after implantation. We encapsulated human ovarian tissue from donors in a dual-layered PEG capsule and implanted these capsules subcutaneously into non-obese diabetic/severe combined immunodeficient gamma (NSG) mice and evaluated follicle survival in fresh and cryopreserved human ovarian tissue that was either encapsulated or non-encapsulated.

## 2 Materials and Methods

### 2.1 Collection of Human Ovarian Tissue

Organ procurement for research purposes followed standardized protocols in place at the International Institute for the Advancement of Medicine (IIAM) and the associated Organ Procurement Organization (OPO) involved in the harvest. For this study ovaries were procured from four deceased donors (age range: 18-26 years) by the IIAM, see **Table 1** for additional donor information. Before cross-clamp, the organs were perfused with either Belzer University of Wisconsin^®^ Cold Storage Solution (Bridge of Life, SC, USA), Custodiol^®^ HTK (Histidine-Tryptophan-Ketoglutarate) Solution (Essential Pharmaceuticals, NC, USA), or SPS-1 Static Preservation Solution (Organ Recovery Systems, IL, USA). Organs were placed in perfusion solution and shipped on ice. De-identified donor information is summarized in **Table 1**, including age, weight, height, BMI, and cross-clamp time (time at which the organ is cut from blood/oxygen supply). Cold ischemic time (CIT) was calculated as the time interval between cross-clamp time of the donor (and subsequent cessation of arterial blood flow to the ovaries) in the operation room and start time of the tissue harvest subsequent to arrival at the laboratory.

**Table 1:** Donor information.

Donor No.	1	2	3	4
**Age (years)**	26	18	18	23
**Height (cm)**	157	160	163	165
**Weight (kg)**	60.2	70.3	52.8	67.9
**BMI**	24.4	27.5	20.0	25.0
**Ethnicity**	Black or African American	Black or African American	White	White
**Cause of Death**	Anoxia	Anoxia	Head trauma	Head trauma
**Storage Solution**	UW	UW	HTK	UW
**Right Ovary Size (cm)**	4x2x1	3x1x0.5	4x3x1.2	4x2.5x1.7
**Left Ovary Size (cm)**	4x2x1	3x1.5x0.5	NA	3.5x2x1.2
**Cardiac arrest/downtime**	YES 30 minutes	NO	NO	YES 10 minutes
Cross-clamp Date and Time*	7/24/2019 16:27	10/14/2019 6:03	1/9/2019 17:22	12/19/2019 15:46
Cold Ischemic Time** (hours)	9.8	18.8	7.9	5.1

UW, University of Wisconsin Solution; HTK, Histidine-Tryptophan-Ketoglutarate Solution.

*Time at which the organ is cut from blood/oxygen supply.

**Time between cross-clamp and the beginning of tissue processing.

### 2.2 Ethical Approval Process

The IIAM procures tissue and organs for non-clinical research from Organ Procurement Organizations (OPOs), which comply with state Uniform Anatomical Gift Acts (UAGA) and are certified and regulated by the Centers for Medicare and Medicaid Services (CMS). These OPOs are members of the Organ Procurement and Transplantation Network (OPTN) and the United Network for Organ Sharing (UNOS) and operate under a set of standards established by the Association of Organ Procurement Organizations (AOPO) and UNOS. Informed, written consent from the deceased donor’s family was obtained for the tissue used in this publication. A biomaterial transfer agreement is in place between IIAM and the authors that restricts the use of the tissue for pre-clinical research that does not involve the fertilization of gametes. The use of deceased donor ovarian tissue in this research is categorized as ‘not regulated’, per 45 CFR 46.102 and the ‘Common Rule’, as it does not involve human subjects and complies with the University of Michigan’s IRB requirements as such.

### 2.3 Tissue processing

**Figure 1:**
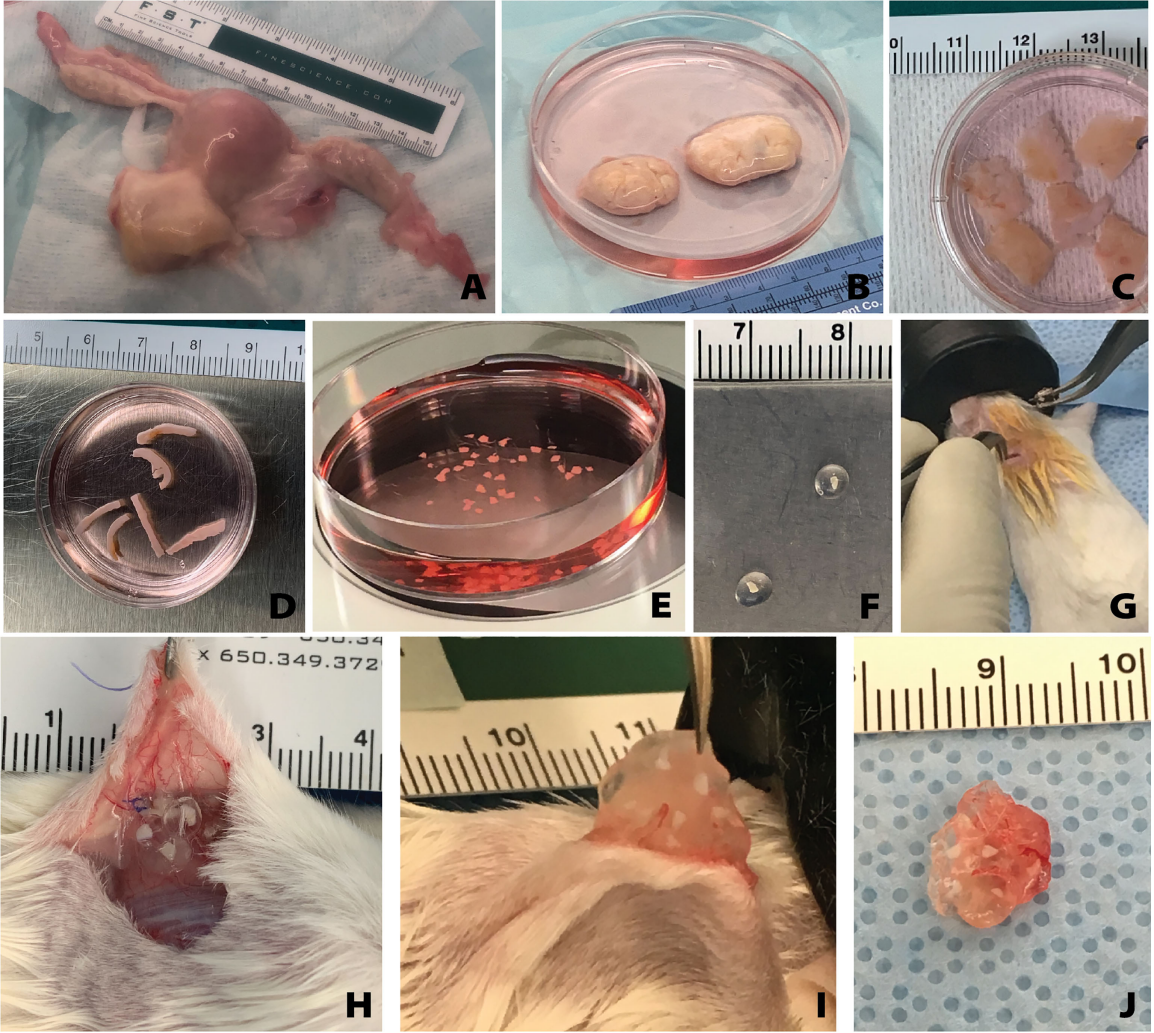
Overview of human tissue processing and implantation. Tissue received from UNOS donors **(A)** was processed to isolate the ovaries **(B)**. Ovarian cortex tissue approximately 10mm x 10mm x 1mm is removed **(C)**, then cut to approximately 1mm x 10mm x 1mm **(D)**, and finally cut to approximately 1mm x 1mm x 1mm **(E)**. The small tissues are encapsulated in the hydrogel capsule **(F)** and implanted subcutaneously **(G)**. Removal of the capsules from the subcutaneous space **(H)** was conducted at various time points. The capsules were removed from the mice **(I, J)** and then fixed for histological processing and staining.

**Figure 2:**
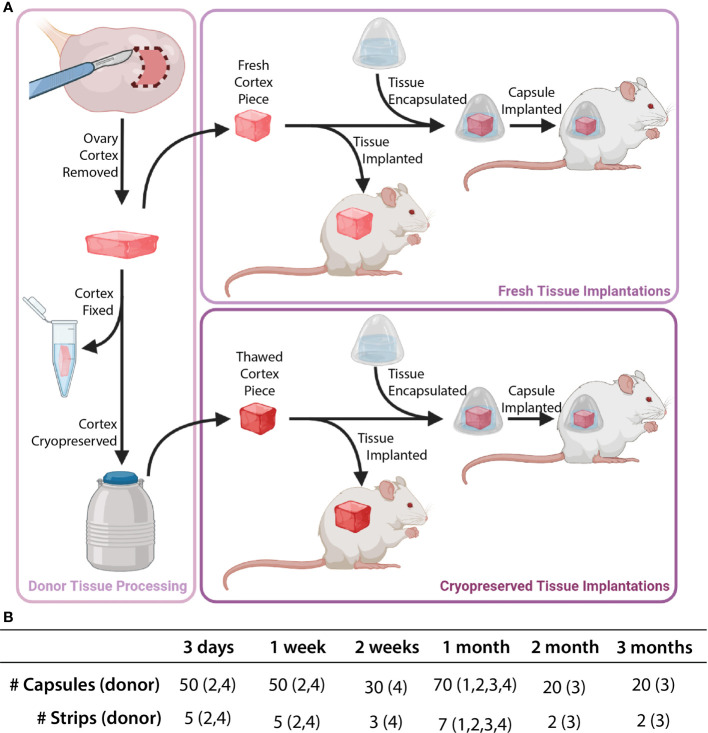
Description of implanted tissue groups. Tissue processing was conducted as shown in part **(A)**. Cortical tissue strips were cut into squares either as fresh tissue (Donors 1 and 2) or post-cryopreservation (Donors 3 and 4). Capsules and control tissue were implanted in mice. The total number of capsules/strips at each time point are shown in part **(B)**.

All tissue processing was done aseptically in a biosafety cabinet. After receiving donor tissue the ovaries were separated from other reproductive tissues (i.e. the uterus, fallopian tubes) (**Figures 1A, B** **)**. The ovaries were decortified using a custom cutting guide (Reprolife Japan, Tokyo) to remove 1 mm thick cortex pieces that were approximately 10mmx10mm squares (**Figures 1C**, **2A**). The squares were then cut into approximately 1mm wide strips, 10mm in length and 1mm thick (**Figures 1D**, **2A**) using a McIlwain Tissue Chopper (The Mickle Laboratory Engineering Co. Ltd., Surrey, UK) and aseptically transferred into holding media (Quinn’s Advantage Medium with HEPES (QAMH), 10% Quinn’s Advantage Serum Protein Substitute (SPS), CooperSurgical, Måløv, Denmark). The tissue was divided into three groups: 1) Encapsulation of fresh tissue pieces followed by immediate implantation in mice; 2) Fixation using Bouin’s fixative (Ricca Chemical, USA) or 4% paraformaldehyde (PFA) (AlfaAesar, USA), and stored overnight at 4°C; 3) Cryopreservation using either slow freezing or vitrification methods.

### 2.4 Cryopreservation

#### 2.4.1 Slow Freezing:

The methods as described by Xu et al. were used for slow freezing (24). Briefly, strips of cortical tissue approximately 1mm x 10mm x 1mm were placed into cryovials (Nunc, Roskilde, Denmark) filled with pre-cooled cryoprotectant media (QAMH, 10% SPS, 0.75M dimethyl sulfoxide (DMSO) (Sigma Aldrich, St. Louis, USA), 0.75M ethylene glycol (Sigma Aldrich, St. Louis, USA), 0.1M sucrose (Sigma Aldrich, St. Louis, USA), and equilibrated at 4°C for at least 30 minutes. After equilibration, cryovials were loaded into the Cryologic Freeze Control System (Cryologic, Victoria, Australia). Vials were then frozen *via* the following protocol: samples were (1) cooled from 4°C to -9°C at a rate of -2°C/min (2) equilibrated for 6 min at -9°C (3) seeded manually using large swabs cooled by submersion in liquid nitrogen (4) held for 4 min at -9°C (5) cooled to -40°C at a rate of -0.3°C/min and (6) plunged into liquid nitrogen and stored in a cryogenic storage dewar until thawed for use.

#### 2.4.2 Vitrification

The methods as described by Kagawa et al. were used for vitrification, with minor changes to the timing (25). Briefly, strips of tissue approximately 1mm x 10mm x 1mm were first transferred to an equilibration solution (7.5% ethylene glycol, 7.5% DMSO, and 20% SPS) for 25 minutes and then transferred to a vitrification solution (20% ethylene glycol, 20% DMSO, 0.5M sucrose, and 20% SPS) until the tissue sank to the bottom of the vial, indicating saturation with the solution. Each strip was placed on a 25 µm thick copper cryostrip (Lyon Industries, South Carolina, USA), which was then submerged in liquid nitrogen for approximately 30 seconds. Strips were transferred into a cryovial (Nunc, Roskilde, Denmark) filled with and submerged in liquid nitrogen. Samples were stored in liquid nitrogen until thawed for use.

### 2.5 Thawing Tissue

#### 2.5.1 Slow Frozen Tissues:

The process described by Xu et al. was followed with minimal changes (24). Briefly, vials with ovarian tissue were removed from liquid nitrogen and placed in a 37°C bath. Once the cryoprotectant media in the vial had thawed, the tissue was removed from the vial and put into Thaw Solution One (1M DMSO, 0.1M Sucrose, 10% SPS in QAMH) for ten minutes. Tissue was then incubated sequentially in Thaw Solution Two (0.5M DMSO, 0.1M Sucrose, 10% SPS in QAMH), Three (0.1M Sucrose, 10% SPS in QAMH), and Four (10% SPS in QAMH) for ten minutes each. All thaw solutions were maintained at room temperature during this process. Tissue strips were cut into cubes measuring approximately 1mm3 while still in Thaw Solution Four.

#### 2.5.2 Vitrified Tissues

The process described by Lee et al. was followed, with minor modifications (26). The cryovials with ovarian cortex strips were removed from liquid nitrogen. The copper supports with tissue were immediately transferred into Thaw Solution One (1mg/mL Human Serum Albumin (HSA) (MilliporeSigma, MA, USA) and 1M sucrose in QAMH). Copper supports were removed, and the tissue was incubated at 37°C for three minutes. Tissue was transferred to Thaw Solution Two (1mg/mL HSA and 0.5M sucrose in QAMH) for 5 minutes at room temperature. Tissue was then transferred to Thaw Solution Three (1mg/mL HSA in QAMH), brought up to 37°C, and cut into cubes measuring approximately 1mm3.

### 2.6 Encapsulation

The pieces of ovarian cortical tissue (1mm^3^) (**Figure 2A** **)** were maintained in the final thaw solution media (Solution 4 for slow freezing, Solution 3 for vitrification) at 37°C until encapsulation (**Figure 1E**). The PEG core was prepared by cross-linking 8-arm PEG-VS (40 kDa, Jenkem Technology, Beijing, China) (5% w/v) with plasmin sensitive peptide (Ac-GCYK↓NSGCYK↓NSCG, MW 1525.69 g/mol, > 90% Purity, Genscript, ↓ indicates the cleavage site of the peptide). The PEG shell was prepared with 4-arm PEG-VS (20 kDa, Jenkem Technology) (5% w/v), Irgacure 2959 (Ciba, Switzerland, MW = 224.3) (0.4% w/v), and N-vinyl-2-pyrrolidone (Sigma-Aldrich, St. Louis, USA) (0.1% v/v).

The tissue was then placed in a 4µL droplet of degradable PEG core pre-cursor solution. After five minutes of crosslinking the core was transferred into 10µL of non-degradable PEG shell pre-cursor solution. The shell was cross-linked *via* UV light at constant intensity (4.4mW/cm^2^, 6 minutes). Encapsulated tissue (**Figures 1F**, **2A**) was maintained in Leibovitz L-15 media (Gibco, USA) at 37°C until implantation.

### 2.7 Subcutaneous Implantation

Animal experiments for this work were performed in accordance with the protocol approved by the Institutional Animal Care and Use Committee (IACUC) at the University of Michigan (PRO00007716 & PRO00009635). The IACUC guidelines for survival surgery in rodents and the IACUC Policy on Analgesic Use in Animals Undergoing Surgery were followed for all procedures.

Female NSG mice (strain 005557, The Jackson Laboratory, Bar Harbor, ME, USA) 6-8 weeks old were anesthetized using isoflurane (2-3%) *via* inhalation. Mice were given preemptive analgesics (Carprofen, RIMADYL, Zoetis, USA, 5mg/kg body) *via* subcutaneous injection. An incision was made in the medial/dorsal skin. Nine to ten PEG capsules, along with an equivalent amount of non-encapsulated tissue (as a control), were inserted into the dorsal subcutaneous space in the mouse (**Figures 1G**, **2B**). The control graft was inserted subcutaneously and sutured using 5/0 absorbable sutures (AD surgical) to the subcutaneous tissue to ensure graft recovery. Using 5/0 absorbable sutures the incision was closed, with special attention paid to avoid suturing capsules/control tissue. Mice recovered in a clean cage and were monitored post-operatively for 7-10 days.

### 2.8 Implant Removal

Mice were anesthetized using isoflurane as described above. An incision was made in the medial/dorsal skin, avoiding implanted grafts. The encapsulated and control grafts were removed (**Figures 1H–J**), placed in either Bouin’s fixative or 4% PFA overnight at 4°C, washed, and stored in 70% ethanol or PBS, depending on respective fixative.

### 2.9 Histology

All samples were processed at the Histology Core in the Dental School at the University of Michigan. The paraffin embedded tissue blocks were serially sectioned at a thickness of 5µm and stained with hematoxylin and eosin.

### 2.10 Follicle Counting

Slides were viewed using a brightfield microscope (Leica DM 1000, Germany) at 20 or 40x magnification. Encapsulated tissue and control non-encapsulated grafts were analyzed for follicle density. Every 16th section was analyzed for follicles for all groups. For slow frozen encapsulated tissue groups between 6 and 17 sections were analyzed per mouse (n=3 mice per time point). For slow frozen non-encapsulated control tissue between 5 and 14 sections were analyzed per mouse (n=3 mice per time point). In the slow frozen groups, each data point represents the follicle density calculated from all capsules retrieved from each mouse. For vitrified encapsulated tissue groups between 4 and 10 sections were analyzed per mouse (n=2 mice per time point). For vitrified non-encapsulated control tissue between 5 and 8 sections were analyzed per mouse (n=2 mice per time point). In the vitrified groups, each data point represents the follicle density calculated from all capsules retrieved from each mouse. For fresh fixed tissue, between 8 and 12 sections were analyzed per donor where each data point represents the follicle density calculated from a single analyzed section. Follicles were counted manually and follicle stage was identified using standard morphological guidelines (27). All primordial and primary follicles were counted for each analyzed section. Preantral and antral were counted only after comparing the location of the follicle in the tissue and follicle size in preceding and subsequent sections to avoid “double-counting”. Density measurements were calculated using an estimated area (1mm^2^ per section) based on the approximate geometry of the sample, and the estimated tissue section thickness. The follicle density values reported in this study are all normalized to the same volume.

### 2.11 Statistical Analysis

Statistical analysis was performed using GraphPad Prism software. A 1-way ANOVA was used for fresh tissue analysis comparing follicle density between donors. Tukey’s comparison test was used for implanted tissue analysis to a evaluate differences in follicle density across two variables (time and encapsulation). The results were considered statistically significant when p < 0.05.

## 3 Results

### 3.1 Fresh Donor Tissue Prior to Processing Reveals Donor Heterogeneity

**Figure 3:**
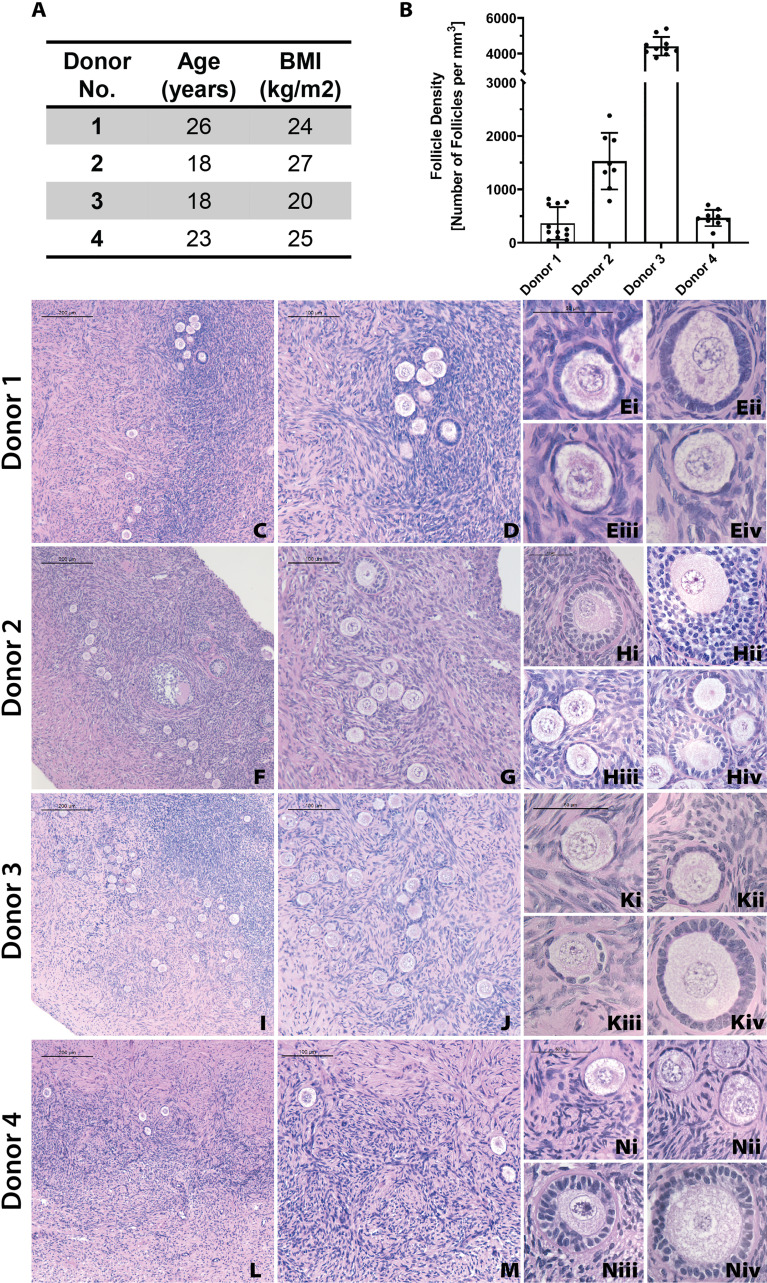
Donor metrics and initial histology. The donor tissues used in this study were selected from a bank from donors with various ages and BMI values **(A)**. Follicle density values were calculated for each donor from fresh/fixed tissues **(B)**. The large differences in average follicle density between donors is supported by histological images **(C–N)**. Representative images from Donor 1 **(C–E)**, Donor 2 **(F–H)**, Donor 3 **(I–K)**, and Donor 4 **(L–N)** show stark differences in follicle distribution at low magnification **(C, F, I, L)**. Increased magnification shows the presence of primordial **(E iii., K i., N i)**, primary **(E i., H iv., K ii.)**, and secondary **(H ii**.**)** follicles. Scale bars represent 200μm for **C**, **F**, **I**, and **L**. Scale bars represent 100 μm for **D**, **G**, **J**, and **M**. Scale bars represent 50μm for all others.

First, we analyzed the follicle density in ovarian cortex from each donor to benchmark a starting point for the grafts. In this study we used ovarian tissue from 4 young healthy deceased donors, ranging from 18 to 26 years old. Inherently, human ovarian cortex varies greatly between donors and location in the ovary. As expected, all donor tissues displayed a high degree of heterogeneity between different strips and various locations in the same cortical strip from the same donor as well as across different donors. The average follicle density in ovarian tissue from Donor 1, 26 years old and BMI of 24.4, was 361 ± 304 follicles/mm^3^ (average ± standard deviation). The average follicle density in the ovarian cortex of Donor 2, 18 years old and BMI 27.5, was 1528 ± 528 follicles/mm^3^. Donor 3, 18 years old and BMI of 20, had an average follicle density of 4414 ± 521 follicles/mm^3^. Donor 4, 25 years old and BMI of 24.9, had an average follicle density of 464 ± 152 follicles/mm^3^ (**Figures 3A, B** **)**. Donor 3 had the greatest follicle density, 4414 ± 521 follicles/mm^3^ (p<0.0001) compared to the other three donors. The follicle density of Donor 2 was greater compared to the lower follicle densities of Donors 1 and 4 (p <0.0001). The follicle densities of Donors 1 and 4 were relatively similar and not significantly different. Histological images from each donor showed multiple primordial and primary follicles (**Figures 3C–N**). The cortical stroma had regions of densely packed primordial follicles with oocytes surrounded by a single layer of flat squamous granulosa cells (**Figure 3E iii.**, **Figure 3K i.**, **Figure 3N i.**). In addition to primordial follicles, primary and small preantral follicles were also identified in the ovarian tissue from all donors. Primary follicles showed the characteristic oocyte surrounded by a single layer of cuboidal granulosa cells (**Figure 3E i.**, **Figure 3H iv.** **Figure 3K ii.**), while preantral follicles had a few layers of granulosa cells surrounding the oocyte (**Figure 3H ii.**).

### 3.2 Follicles in Encapsulated Fresh Ovarian Cortical Tissues Survive for At Least One Month After Implantation in NSG Mice

**Figure 4:**
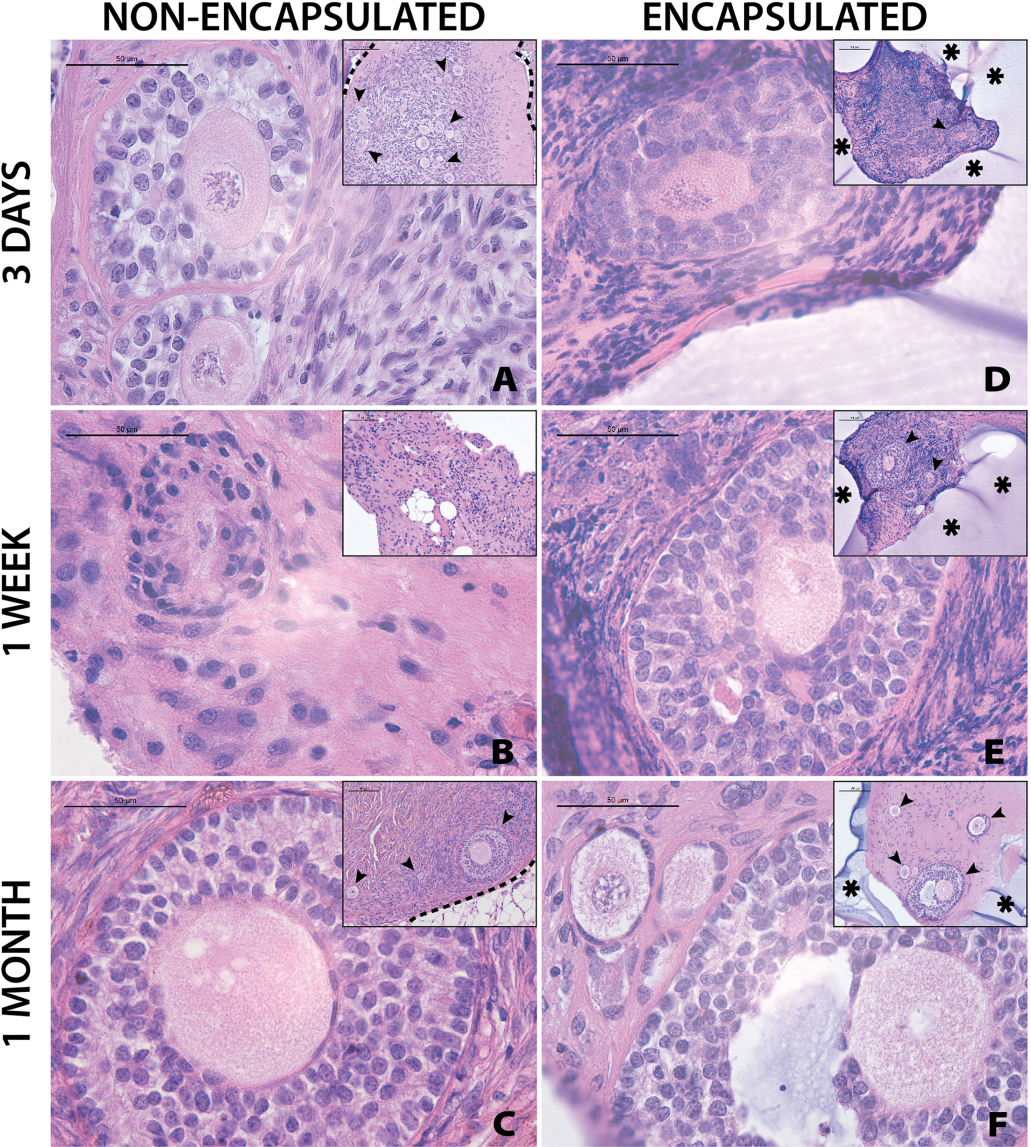
Follicles in fresh tissue survive up to one month. Representative images from non-encapsulated fresh tissue samples **(A–C)** and encapsulated fresh tissue samples **(D–F)** are shown here. These samples were removed from mice at various time points of 3 days **(A, D)**, 1 week **(B, E)**, and 1 month **(C, F)**. Scale bars represent 100μm for inserts and 50μm for all others. Black arrow heads (➤) indicate follicles, asterisks (_*_) indicate the PEG capsule, and dashed black lines (—) indicate the human ovarian tissue border with surrounding murine tissue after implantation.

Grafting of non-encapsulated human ovarian tissue is a gold standard for restoration of fertility in human patients and animal models ( (28)). However, whether encapsulated human ovarian allograft survives encapsulation and implantation has yet to be demonstrated. Here, we investigated whether the encapsulation of fresh ovarian cortical tissue in a hydrogel decreases follicle survival and longevity after implantation in mice, and compared to non-encapsulated fresh tissue. Immediately after receiving and processing ovaries from Donors 1 and 2, we encapsulated and implanted ovarian cortical tissues in mice with nine to ten capsules per animal for time periods of 3 days (n=2 mice), 1 week (n=2), and 1 month (n=3) (**Figures 4D–F** respectively). A non-encapsulated cortical strip measuring 1mm x 10mm x 1mm was implanted in each mouse and served as a control (**Figures 4A–C**). Recovery of the capsules from the subcutaneous space was reasonably straightforward as the capsules typically clumped together (**Figure 1J**). Eight to ten capsules were recovered in six out of seven mice (no capsules were recovered from one animal, possibly due to degradation). Non-encapsulated control tissue was recovered in all seven mice. Eight to twenty-six sections for each implant type (encapsulated or non-encapsulated) were analyzed using bright field microscopy to assess follicle survival. Multiple follicles at different stages ranging from primordial to antral were present in retrieved encapsulated tissue from all time points up to a month post implantation within the same capsule (**Figure 4F**). Nuclear staining of the encapsulated tissue retrieved after 3 days and 1 week identified the presence of multiple stromal cells around the follicles. The encapsulated tissue had multiple surviving follicles after 1 month, however qualitative analysis showed a decrease in number of stromal cells. Overall, we concluded that follicle survival in human ovarian cortex was not decreased by the encapsulation process and was similar to the non-encapsulated controls.

### 3.3 Slow Frozen, Short-Term Implanted Tissues Tolerate Encapsulation and Survive to the Same Extent as Non-Encapsulated Tissue Following Implantation

**Figure 5:**
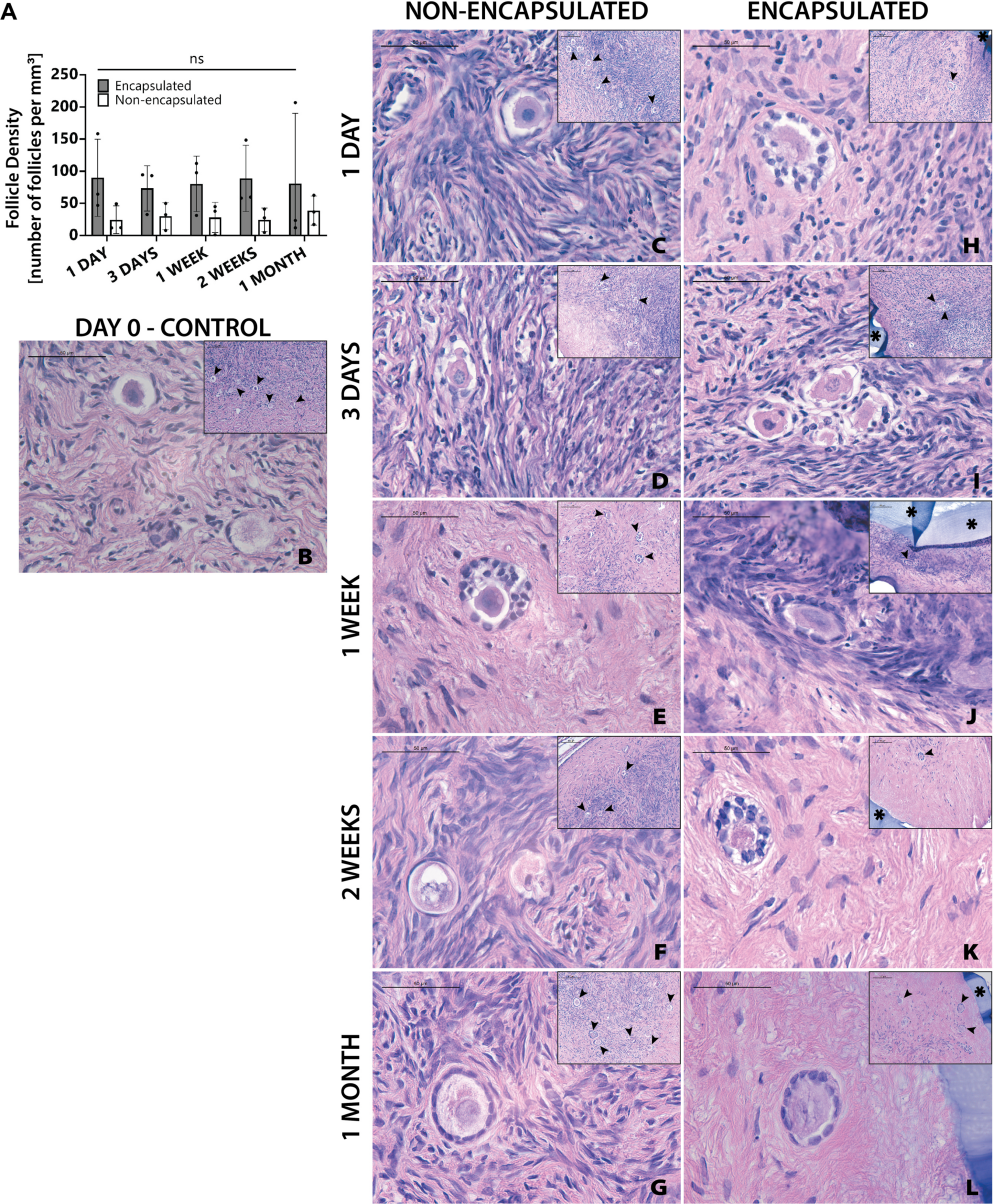
Follicles in slow frozen tissue survive up to one month. Follicle density values **(A)** are determined from histological images **(C–L)**. Non-implanted thawed/fixed tissue **(B)** is a control for non-encapsulated groups **(C–G)** and encapsulated groups **(F–L)**. These samples were removed from mice at various time points of 1 day **(C, H)**, 3 days **(D, I)**, 1 week **(E, J)**, 2 weeks **(F, K)**, and 1 month **(G, L)**. Scale bars represent 100μm for inserts and 50μm for all others. Black arrow heads (➤) indicate follicles, asterisks (_*_) indicate the PEG capsule, and dashed black lines (—) indicate the human ovarian tissue border with surrounding murine tissue after implantation.

The next objective was to determine whether encapsulation of cryopreserved ovarian tissue using the slow freezing approach, similar to the current clinical practice recommended for fertility preservation in pediatric patients, negatively affected follicle survival. Ovarian cortical strips from Donor 4 that had previously been slow frozen, were thawed and encapsulated. Multiple capsules and all non-encapsulated tissues were recovered in all 20 mice. There was no statistically significant difference in follicle density between encapsulated and non-encapsulated grafts at any time point (**Figure 5A**). For the 1-day time point, the follicle density was 24 ± 21 follicles/mm^3^ (average ± standard deviation) in the non-encapsulated graft and 90 ± 60 in the encapsulated tissue. For the 3-days’ time point, the average follicle density was 30 ± 21 and 73 ± 35, for the 1-week time point, the average follicle density was 28 ± 23 and 80 ± 43, for the 2-weeks’ time point, the average follicle density was 25 ± 18 and 89 ± 51 for the 1-month time point, the average follicle density was 39 ± 22 and 81 ± 109, respectively. The difference in follicle density was not statistically significant between different time points, nor was it different when comparing between encapsulated and non-encapsulated groups at each time point. These findings suggest that after slow freezing (1) human ovarian follicles tolerate encapsulation in the dual-layered PEG hydrogel capsule and (2) encapsulated human ovarian tissue survives to the same extent as non-encapsulated tissues when implanted in mice.

Although follicle numbers were not significantly different between groups, we also wanted to probe whether there were any differences between groups in terms of the quality of the stromal compartment. Qualitative histological analysis of tissue fixed immediately post-thawing (**Figure 5B**) and tissue fixed after implantation showed similar distribution of stromal cells (marked with hematoxylin). Furthermore, similar stromal cell densities were evident across time points, as well as between encapsulated (**Figures 5H–L**) and non-encapsulated groups (**Figures 5C–G**) at the same time points, except for the 1-month encapsulated tissue (**Figure 5L**). The 1-month encapsulated tissue had less cellular staining around the follicles and more fibrous portions of the extracellular matrix (stained pink with eosin). Furthermore, the types of follicles seen in encapsulated and non-encapsulate groups at various time points were comparable, with primordial and primary follicles being the most prevalent. Taken together, these observations support the hypothesis that follicles can survive slow freezing before encapsulation and survive up to one month *in vivo*.

### 3.4 Vitrified, Long-Term Implanted Tissues Tolerate Encapsulation and Survive Up to Three Months *In Vivo*

**Figure 6:**
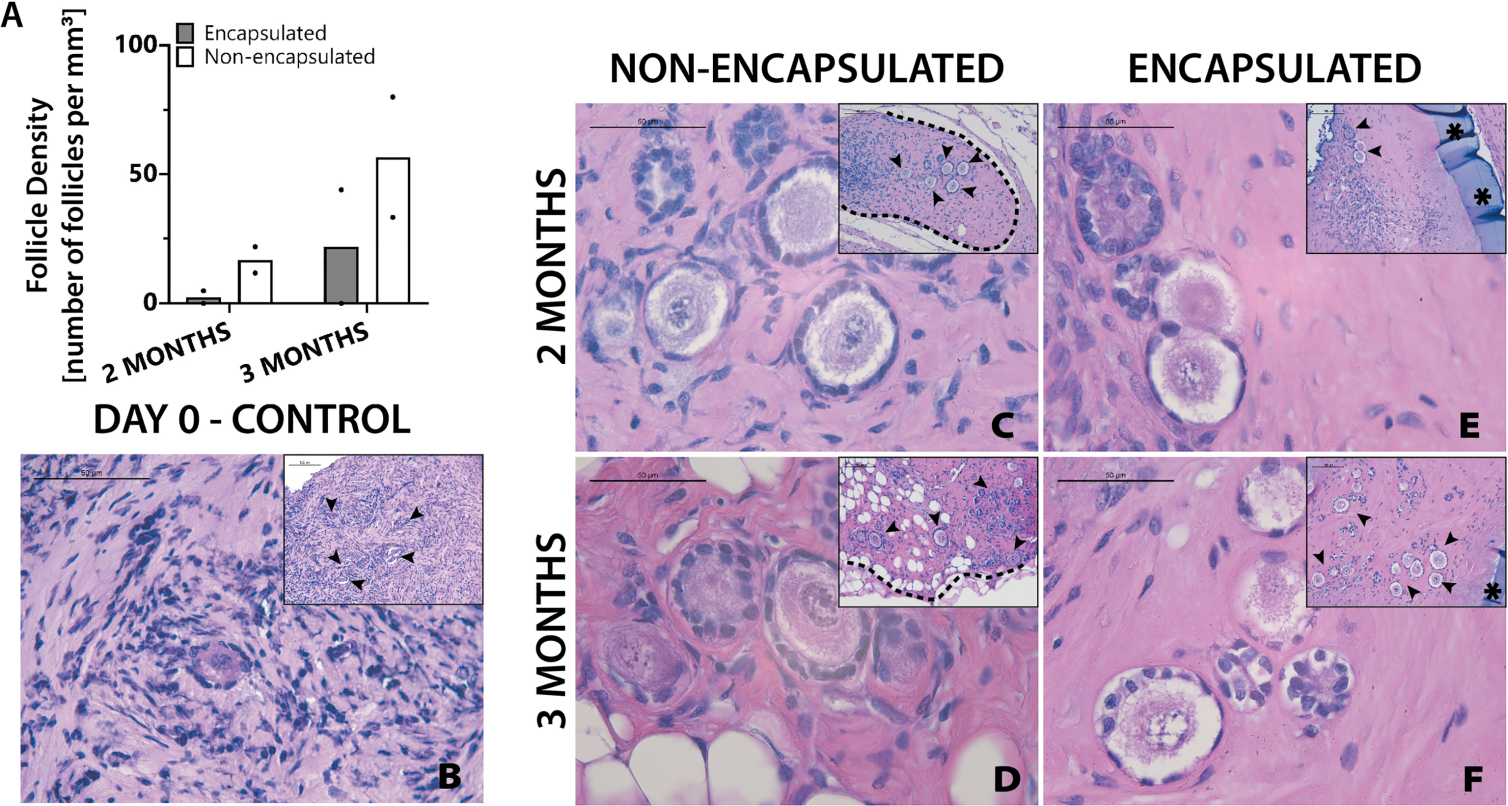
Follicles in vitrified tissue survive up to three months. Follicle density values **(A)** are determined from histological images **(C–F)**. Non-implanted thawed/fixed tissue **(B)** is a control for non-encapsulated groups **(C, D)** and encapsulated groups **(E, F)**. These samples were removed from mice at various time points of 2 months **(C, E)** and 3 months **(D, F)**. Scale bars represent 100μm for inserts and 50μm for all others. Black arrow heads (➤) indicate follicles, asterisks (_*_) indicate the PEG capsule, and dashed black lines (—) indicate the human ovarian tissue border with surrounding murine tissue after implantation.

The final objective of this study was to investigate whether the two clinically available cryopreservation methods, slow freezing and vitrification specifically, have different outcomes of follicle survival in grafted tissue following encapsulation and implantation. Vitrified tissue from Donor 3 was encapsulated and implanted in mice for 2 and 3 months. Non-encapsulated, 1mm^3^ grafts served as controls. Two months after implantation the average follicle density was 17 follicles/mm^3^ in the non-encapsulated grafts and 2 follicles/mm^3^ in the encapsulated tissue. For the 3-month time point, the average follicle density was 57 follicles/mm^3^ in the non-encapsulated graft and 22 follicles/mm^3^ in the encapsulated tissue (**Figure 6A**). Vitrified tissue had greater cell density in the stroma (**Figure 6B**) than frozen tissue before implantation (**Figure 5B**). Encouragingly, follicles in implanted tissues survived up to three months post-implantation, but the surrounding stroma had lower cell density compared to tissue prior to implantation (**Figures 6C–F**); the decrease in stroma cell density was observed in all implanted groups, similarly to the one-month time point for encapsulated slow frozen tissue (**Figure 5L**) In comparison to other slow frozen tissue, vitrified groups similarly have a follicle pool comprised predominantly of primordial and primary follicles. Based on our findings, we concluded that follicles and ovarian stromal cells tolerate vitrification before encapsulation, and survive up to three months *in vivo*.

## 4 Discussion

Previously, using PEG-based immunoisolating capsules we have demonstrated that the capsule protected murine ovarian allografts in immune competent and sensitized murine hosts and promoted folliculogenesis up to antral stages after single and repeated implantations (10). Despite mice and humans having similar hypothalamus-pituitary-gonad axis regulation, circulating cytokines and hormones, and developmental stages of follicular differentiation, existence of other physiological and anatomical differences between human and murine ovarian tissues necessitates further investigation to determine translatability of the use of immunoisolating capsules from mouse to human tissues. One example of the challenges working with human tissue is that human primordial follicles are spread heterogeneously in the stroma of ovarian cortex and require normalization of the follicle counts in each graft. The heterogeneity of human tissue introduced significant variance into our quantification of follicle density. To control for this, encapsulated tissues and corresponding non-encapsulated tissue originating from the same donor were included within same groups to minimize the impact of inherent heterogeneity between donors. Tissue pieces placed in the encapsulated groups and non-encapsulated groups were randomized to minimize skewing of results due to heterogeneity present in a single ovary. Even with these adaptations it is uncertain if differences seen are an artifact of tissue heterogeneity or are a result of tissue treatment; persistence of a large variability resulting in largely qualitative methods of analysis. One option to minimize the effect of heterogeneity on follicle density analysis is to significantly increase the number of human donors and recipient mice. Another option is to use cryopreserved tissues that would have undergone a thorough characterization prior to xenografting, allowing for donors with similar follicle densities to be used in the same study.

Ovarian tissue cryopreservation (OTC) enables preservation of many thousands of primordial follicles all at once without the need of ovarian stimulation and can be performed at any age from early prepubertal years to late thirties. Slow freezing and vitrification of ovarian tissues are the two most studied methods of cryopreservation. The majority of clinical data, such as the rates of live births (29–31), the efficiency and safety of OTC in terms of follicle survival and patients’ outcomes after transplantation, is available for slow freezing methods, which was considered experimental for a few decades and was recently clinically approved (32). Slow freezing in general uses a low amount of cryoprotective agents to reduce cell toxicity. This method utilizes standardized equipment and detailed cooling protocols such that there is decreased variability between operators, as well as between batches. On the downside, slow freezing poses the risk of intracellular ice crystal formation, which can be minimized by manual seeding at the media/air interface (33, 34). Vitrification, another cryopreservation method, is currently only approved for embryo and egg cryopreservation (35). It utilizes greater concentrations of cryoprotectants that prevent ice crystal formation. Vitrification does not require standardized equipment, which results in some variability between operators and batches. So far, this method has been clinically approved for oocyte and embryo cryopreservation but is still considered experimental for OTC. Possibly, in the future, it may become clinically approved for OTC as well.

Both cryopreservation methods have been shown to have negligible impact on follicle count as compared to fresh tissue (36) and ensure the survival of primordial follicles, which are critical for the application described in this study; dormant primordial follicles in the implanted encapsulated tissue functions as the ovarian reserve, determining the quality and the lifespan of the encapsulated tissue. Additionally, OTC allows important screening for disease or infection and analysis of the follicle density in the donor tissue that would not be possible with fresh tissue. While some reports show that vitrification results in (1) decreased primordial follicle DNA damage and (2) better outcomes for stromal tissue (37), others indicate that slow freezing is superior for (1) preserving primordial follicles, (2) preventing DNA damage, and (3) promoting follicular cell proliferation (26). Comparison of the survival of slow frozen and vitrified tissues with fresh tissues used as controls showed fresh tissues to have more mature (i.e. secondary/antral) follicles, as expected. More mature follicles contain oocytes that are more metabolically active and antral follicles contain the cumulus-oocyte complex, resulting in decreased cryoprotectant agent penetration and therefore increased probability of ice crystal formation (38). Our observation that the stroma in slow frozen tissues (**Figure 5**) is less fibrous as compared to vitrified tissues (**Figure 6**) is somewhat unexpected since the literature indicates that vitrification typically has better stromal cell survival (37). The most likely explanation for these differing results is that there was more variability between operators during the vitrification process. Notably, an important finding from the present study is that follicles can survive the process of cryopreservation and survive for at least 30 days *in vivo*, regardless of cryopreservation method.

Encapsulation may slow down the diffusion of nutrients, yet it is necessary to prevent rejection of the grafts. Our comparison of tissues that underwent the encapsulation process to tissues that did not, found that the follicle densities of non-encapsulated tissues were not higher than the follicle densities in encapsulated tissues. Qualitative examination of the implants also indicates minimal differences between encapsulated and non-encapsulated tissues at early time points, but larger differences begin to appear starting around one month. The main change observed is a decrease in stromal cell density in encapsulated tissues. This trend is emphasized by greater areas of fibrous stromal extracellular matrix, the mostly eosin-stained matrix without visible nuclei. The relative similarities between short term implants and subsequent differences in long term implants indicate that the process of encapsulation is not harmful to human tissues. It is hypothesized that as time goes on the rate of diffusion of nutrients is inadequate to support these tissues.

In conclusion, we have shown that an immunoisolating dual PEG capsule supported follicle survival in human ovarian xenografts up to 90 days *in vivo*. The process of encapsulation did not decrease follicle density compared with non-encapsulated grafts. Cryopreservation of ovarian cortical strips allowed for thorough analysis of follicular density to ensure graft longevity. Moving forward towards clinical translation of this technology, a greater scale of characterization of the donor tissue and optimization of the PEG shell to increase diffusion and increase stromal cell survival at later time points, should be done.

## Data Availability Statement

The raw data supporting the conclusions of this article will be made available by the authors, without undue reservation.

## Ethics Statement

The animal study was reviewed and approved by Institutional Animal Care and Use Committee at the University of Michigan.

## Author Contributions

Study design: MB, AS. Murine experiments: MB, HK, JD. Histological sample preparation: MB, PH. Follicle counting and figure compilation: MB. Wrote original manuscript draft: MB, CF, VP, AS. Manuscript review & editing: MB, HK, PH, CF, JD, MC, VP, AS. All authors contributed to the article and approved the submitted version.

## Funding

National Institutes of Health grant R01HD104173, R01EB022033 (MB, CF, JD, MC, AS). National Institutes of Health grant T32DE007057 (MB). National Institutes of Health grant R01HD09823 (HK, PH, AS). National Institutes of Health grant F30HD100163 (HK). Chan Zuckerberg Initiative (CF, MC, VP, AS).

## Conflict of Interest

The authors declare that the research was conducted in the absence of any commercial or financial relationships that could be construed as a potential conflict of interest.

The handling editor YF declared a past collaboration with the author AS.

## Publisher’s Note

All claims expressed in this article are solely those of the authors and do not necessarily represent those of their affiliated organizations, or those of the publisher, the editors and the reviewers. Any product that may be evaluated in this article, or claim that may be made by its manufacturer, is not guaranteed or endorsed by the publisher.

## References

[1] Jones RM Pattwell SS. Future Considerations for Pediatric Cancer Survivorship: Translational Perspectives From Developmental Neuroscience. Dev Cognit Neurosci (2019) 38:100657. doi: 10.1016/j.dcn.2019.100657 31158802 PMC6697051

[2] Siegel RL Miller KD Fuchs HE Jemal A. Cancer Statistics, 2021. CA Cancer J Clin (2021) 71:7–33. doi: 10.3322/caac.21654 33433946

[3] Akasha AM Woodruff TK. Oncofertility: Preservation of Ovarian Function After a Cancer Diagnosis. 3rd ed. Leung PCK Adashi EY, editors. London: Elsevier Inc (2019) p. 501–8. doi: 10.1016/B978-0-12-813209-8.00031-5

[4] International Programme on Chemical Safety. “Endocrinology and Endocrine Toxicology”. In: Damstra T Barlow S Bergman A Kavlock R van der Kraak G, editors. Global Assessment of the State-Of-the-Science of Endocrine Disruptors. Geneva: World Health Organization (2002). p. 11–32.

[5] Pierson RA. “Human Folliculogenesis Revisited: The Menstrual Cycle Visualized by Ultrasonography”. In: Leung PCK Adashi EY, editors. The Ovary. London: Elsevier Inc (2019). p. 51–69. doi: 10.1016/b978-0-12-813209-8.00003-0

[6] McCartney CR Marshall JC. “Neuroendocrinology of Reproduction”. In: Yen & Jaffe’s Reproductive Endocrinology: Physiology, Pathophysiology, and Clinical Management. Philadelphia: Elsevier Inc (2019). p. 1–24.e8. doi: 10.1016/B978-0-323-47912-7.00001-9

[7] Langer RD. The Evidence Base for HRT: What can We Believe? Climacteric (2017) 20:91–6. doi: 10.1080/13697137.2017.1280251 28281363

[8] Christin-Maitre S. Use of Hormone Replacement in Females With Endocrine Disorders. Horm Res Paediatr (2017) 87:215–23. doi: 10.1159/000457125 28376481

[9] Day JR David A Cichon AL Kulkarni T Cascalho M Shikanov A. Immunoisolating Poly(Ethylene Glycol) Based Capsules Support Ovarian Tissue Survival to Restore Endocrine Function. J BioMed Mater Res Part A (2018) 106:1381–9. doi: 10.1002/jbm.a.36338 PMC5874172 29318744

[10] Day JR David A Barbosa MG de M Brunette MA Cascalho M Shikanov A. Encapsulation of Ovarian Allograft Precludes Immune Rejection and Promotes Restoration of Endocrine Function in Immune-Competent Ovariectomized Mice. Sci Rep (2019) 9:16614. doi: 10.1038/s41598-019-53075-8 31719632 PMC6851353

[11] Day JR David A Kim J Farkash EA Cascalho M Milašinović N. The Impact of Functional Groups of Poly(Ethylene Glycol) Macromers on the Physical Properties of Photo-Polymerized Hydrogels and the Local Inflammatory Response in the Host. Acta Biomater (2018) 67:42–52. doi: 10.1016/j.actbio.2017.12.007 29242160 PMC5794611

[12] Xu J Lawson MS Yeoman RR Molskness TA Ting AY Stouffer RL. Fibrin Promotes Development and Function of Macaque Primary Follicles During Encapsulated Three-Dimensional Culture. Hum Reprod (2013) 28:2187–200. doi: 10.1093/humrep/det093 PMC3712659 23608357

[13] Pangas SA Saudye H Shea LD Woodruff TK. Novel Approach for the Three-Dimensional Culture of Granulosa Cell-Oocyte Complexes. Tissue Eng (2003) 9:1013–21. doi: 10.1089/107632703322495655 14633385

[14] Rios PD Kniazeva E Lee HC Xiao S Oakes RS Saito E. Retrievable Hydrogels for Ovarian Follicle Transplantation and Oocyte Collection. Biotechnol Bioeng (2018) 115:2075–86. doi: 10.1002/bit.26721 PMC6045426 29704433

[15] Vanacker J Luyckx V Dolmans MM Des Rieux A Jaeger J Van Langendonckt A. Transplantation of an Alginate-Matrigel Matrix Containing Isolated Ovarian Cells: First Step in Developing a Biodegradable Scaffold to Transplant Isolated Preantral Follicles and Ovarian Cells. Biomaterials (2012) 33:6079–85. doi: 10.1016/j.biomaterials.2012.05.015 22658800

[16] Shikanov A Zhang Z Xu M Smith RM Rajan A Woodruff TK. Fibrin Encapsulation and Vascular Endothelial Growth Factor Delivery Promotes Ovarian Graft Survival in Mice. Tissue Eng - Part A (2011) 17:3095–104. doi: 10.1089/ten.tea.2011.0204 PMC3226061 21740332

[17] Kniazeva E Hardy AN Boukaidi SA Woodruff TK Jeruss JS Shea LD. Primordial Follicle Transplantation Within Designer Biomaterial Grafts Produce Live Births in a Mouse Infertility Model. Sci Rep (2015) 5:1–11. doi: 10.1038/srep17709 PMC4668556 26633657

[18] Shikanov A Xu M Woodruff TK Shea LD. Interpenetrating Fibrin-Alginate Matrices for *In Vitro* Ovarian Follicle Development. Biomaterials (2009) 30:5476–85. doi: 10.1016/j.biomaterials.2009.06.054 PMC2906124 19616843

[19] Shikanov A Smith RM Xu M Woodruff TK Shea LD. Hydrogel Network Design Using Multifunctional Macromers to Coordinate Tissue Maturation in Ovarian Follicle Culture. Biomaterials (2011) 32:2524–31. doi: 10.1016/j.biomaterials.2010.12.027 PMC3040241 21247629

[20] Tomaszewski CE Constance E Lemke MM Zhou H Padmanabhan V Arnold KB. Adipose-Derived Stem Cell-Secreted Factors Promote Early Stage Follicle Development in a Biomimetic Matrix. Biomater Sci (2019) 7:571–80. doi: 10.1039/c8bm01253a PMC6351215 30608082

[21] Myers M Britt KL Wreford NGM Ebling FJP Kerr JB. Methods for Quantifying Follicular Numbers Within the Mouse Ovary. Reproduction (2004) 127:569–80. doi: 10.1530/rep.1.00095 15129012

[22] Schmidt KLT Byskov AG Andersen AN Müller J Andersen CY. Density and Distribution of Primordial Follicles in Single Pieces of Cortex From 21 Patients and in Individual Pieces of Cortex From Three Entire Human Ovaries. Hum Reprod (2003) 18:1158–64. doi: 10.1093/humrep/deg246 12773440

[23] Griffin J Emery BR Huang I Peterson CM Carrell DT. Comparative Analysis of Follicle Morphology and Oocyte Diameter in Four Mammalian Species (Mouse, Hamster, Pig, and Human). J Exp Clin Assist Reprod (2006) 3:2. doi: 10.1186/1743-1050-3-2 16509981 PMC1413548

[24] Xu M Banc A Woodruff TK Shea LD. Secondary Follicle Growth and Oocyte Maturation by Culture in Alginate Hydrogel Following Cryopreservation of the Ovary or Individual Follicles. Biotechnol Bioeng (2009) 103:378–86. doi: 10.1002/bit.22250 PMC2778231 19191350

[25] Kagawa N Kuwayama M Nakata K Vajta G Silber S Manabe N. Production of the First Offspring From Oocytes Derived From Fresh and Cryopreserved Pre-Antral Follicles of Adult Mice. Reprod BioMed Online (2007) 14:693–9. doi: 10.1016/S1472-6483(10)60670-0 17579980

[26] Lee S Ryu K Kim B Kang D Kim YY Kim T. Comparison Between Slow Freezing and Vitrification for Human Ovarian Tissue Cryopreservation and Xenotransplantation. Int J Mol Sci (2019) 20:3346. doi: 10.3390/ijms20133346 PMC6651588 31288388

[27] Gougeon A. Dynamics of Follicular Growth in the Human: A Model From Preliminary Results. Hum Reprod (1986) 1:81–7. doi: 10.1093/oxfordjournals.humrep.a136365 3558758

[28] Duncan FE Zelinski M Gunn AH Pahnke JE O’Neill CL Songsasen N. Ovarian Tissue Transport to Expand Access to Fertility Preservation: From Animals to Clinical Practice. Reproduction (2016) 152:R201–10. doi: 10.1530/REP-15-0598 PMC5088055 27492079

[29] Donnez J Dolmans M-M. Transplantation of Ovarian Tissue. Best Pract Res Clin Obstet Gynaecol (2014) 28:1188–97. doi: 10.1016/j.bpobgyn.2014.09.003 25450187

[30] Stoop D Cobo A Silber S. Fertility Preservation for Age-Related Fertility Decline. Lancet (2014) 384:1311–9. doi: 10.1016/s0140-6736(14)61261-7 25283572

[31] Gellert SE Pors SE Kristensen SG Bay-Bjørn AM Ernst E Yding Andersen C. Transplantation of Frozen-Thawed Ovarian Tissue: An Update on Worldwide Activity Published in Peer-Reviewed Papers and on the Danish Cohort. J Assist Reprod Genet (2018) 35:561–70. doi: 10.1007/s10815-018-1144-2 PMC5949119 29497953

[32] Nahata L Woodruff TK Quinn GP Meacham LR Chen D Appiah LC. Ovarian Tissue Cryopreservation as Standard of Care: What Does This Mean for Pediatric Populations? J Assist Reprod Genet (2020) 37:1323–6. doi: 10.1007/s10815-020-01794-7 PMC7311630 32390071

[33] Huang H Zhao G Zhang Y Xu J Toth TL He X. Predehydration and Ice Seeding in the Presence of Trehalose Enable Cell Cryopreservation. ACS Biomater Sci Eng (2017) 3:1758–68. doi: 10.1021/acsbiomaterials.7b00201 PMC5558192 28824959

[34] Isachenko V Isachenko E Reinsberg J Montag M Braun F van der Ven H. Cryopreservation of Human Ovarian Tissue: Effect of Spontaneous and Initiated Ice Formation. Reprod BioMed Online (2008) 16:336–45. doi: 10.1016/S1472-6483(10)60593-7 18339253

[35] The Practice Committees of the American Society for Reproductive Medicine and the Society for Assisted Reproductive Technology. Mature Oocyte Cryopreservation: A Guideline. Fertil Steril (2013) 99:37–43. doi: 10.1016/j.fertnstert.2012.09.028 23083924

[36] Fan Y Flanagan CL Brunette MA Jones AS Baker BM Silber SJ. Fresh and Cryopreserved Ovarian Tissue From Deceased Young Donors Yields Viable Follicles. F&S Sci (2021) 2:248–58. doi: 10.1016/j.xfss.2021.06.003 PMC8823279 35146457

[37] Shi Q Xie Y Wang Y Li S. Vitrification Versus Slow Freezing for Human Ovarian Tissue Cryopreservation: A Systematic Review and Meta-Anlaysis. Sci Rep (2017) 7:1–9. doi: 10.1038/s41598-017-09005-7 28819292 PMC5561141

[38] Leonel ECR M Lucci C A. Amorim C. “Cryopreservation of Preantral Follicles”. In: Cryopreservation Biotechnology in Biomedical and Biological Sciences. London: IntechOpen (2018). p. 71–89. doi: 10.5772/intechopen.79538

